# A canine chimeric monoclonal antibody targeting PD-L1 and its clinical efficacy in canine oral malignant melanoma or undifferentiated sarcoma

**DOI:** 10.1038/s41598-017-09444-2

**Published:** 2017-08-21

**Authors:** Naoya Maekawa, Satoru Konnai, Satoshi Takagi, Yumiko Kagawa, Tomohiro Okagawa, Asami Nishimori, Ryoyo Ikebuchi, Yusuke Izumi, Tatsuya Deguchi, Chie Nakajima, Yukinari Kato, Keiichi Yamamoto, Hidetoshi Uemura, Yasuhiko Suzuki, Shiro Murata, Kazuhiko Ohashi

**Affiliations:** 1 Department of Disease Control, Graduate School of Veterinary Medicine, Hokkaido University, Sapporo, 060-0818 Japan; 2 Veterinary Teaching Hospital, Graduate School of Veterinary Medicine, Hokkaido University, Sapporo, 060-0819 Japan; 3 North Lab, Sapporo, 003-0027 Japan; 4 Department of Diagnostic Pathology, Graduate School of Veterinary Medicine, Hokkaido University, Sapporo, 060-0818 Japan; 5 Research Center for Zoonosis Control, Hokkaido University, Sapporo, 001-0020 Japan; 6 Global Station for Zoonosis Control, Global Institution for Collaborative Research and Education (GI-CoRE), Hokkaido University, Sapporo, 001-0020 Japan; 7 Department of Antibody Drug Development, Graduate School of Medicine, Tohoku University, Sendai, 980-8575 Japan; 8 Project of Antibody Drug Development, New Industry Creation Hatchery Center, Sendai, 980-8575 Japan; 9 Research and Development Center, Fuso Pharmaceutical Industries, Ltd, Osaka, 536-0025 Japan

## Abstract

Immunotherapy targeting immune checkpoint molecules, programmed cell death 1 (PD-1) and PD-ligand 1 (PD-L1), using therapeutic antibodies has been widely used for some human malignancies in the last 5 years. A costimulatory receptor, PD-1, is expressed on T cells and suppresses effector functions when it binds to its ligand, PD-L1. Aberrant PD-L1 expression is reported in various human cancers and is considered an immune escape mechanism. Antibodies blocking the PD-1/PD-L1 axis induce antitumour responses in patients with malignant melanoma and other cancers. In dogs, no such clinical studies have been performed to date because of the lack of therapeutic antibodies that can be used in dogs. In this study, the immunomodulatory effects of c4G12, a canine-chimerised anti-PD-L1 monoclonal antibody, were evaluated *in vitro*, demonstrating significantly enhanced cytokine production and proliferation of dog peripheral blood mononuclear cells. A pilot clinical study was performed on seven dogs with oral malignant melanoma (OMM) and two with undifferentiated sarcoma. Objective antitumour responses were observed in one dog with OMM (14.3%, 1/7) and one with undifferentiated sarcoma (50.0%, 1/2) when c4G12 was given at 2 or 5 mg/kg, every 2 weeks. c4G12 could be a safe and effective treatment option for canine cancers.

## Introduction

Cancer is a common cause of death in dogs^1^. Current treatment for dog cancers includes surgery, chemotherapy and radiotherapy. In addition to these, some molecular-targeted therapies including tyrosine kinase inhibitors are available for treatment of a limited number of cancer types such as mast cell tumour^2^. Many attempts have been made to find new options for dog cancer treatment and some targeted antibodies have been developed and tested *in vitro* and *in vivo* ^3, 4^.

In human cancer treatment, immunotherapy has gained attention partially due to the success in some agents targeting immune checkpoint molecules such as programmed cell death 1 (PD-1) and cytotoxic T-lymphocyte-associated protein 4 (CTLA4)^5, 6^. Of particular note, anti-PD-1 antibody has shown promising anticancer activity with tolerable toxicity profiles in a number of clinical trials^5, 7^, altering the standard of treatment for melanoma, non-small cell lung cancer and other cancers. However, there is no such immunotherapy available to date for treatment of dog cancers, limiting treatment options particularly when systemic therapy is needed to control metastatic disease.

PD-1 is a costimulatory receptor expressed mainly on activated T cells. It suppresses T-cell activation upon binding to its ligands, PD-ligand 1 (PD-L1) and PD-L2. In humans, the expression of PD-1 is upregulated in tumour antigen–specific T cells^8^, and aberrant PD-L1 expression in tumour cells or other cells in the tumour microenvironment has been demonstrated in various cancer types^9, 10^. Blocking the PD-1/PD-L1 pathway with anti-PD-1 or PD-L1 antibody restores multiple effector functions of antigen–specific T cells^8, 11^, with subsequent remission of cancer^12^. This approach is considered a promising immunotherapy that targets the immune evasion mechanisms of cancer cells. In dogs, PD-L1 expression was observed in various cancer types including oral malignant melanoma (OMM), hemangiosarcoma and osteosarcoma. PD-1 was significantly upregulated in tumour-infiltrating lymphocytes obtained from OMM^13^. However, the therapeutic potential of the PD-1/PD-L1 blockade has not yet been fully elucidated because of the lack of therapeutic antibody against these molecules that can be used in clinical experiments with dogs.

Recently, rat monoclonal antibodies (mAbs) which recognise dog PD-L1 have been established in our laboratory^14, 15^. For clinical purposes, rat mAb needs to be canine-chimerised to reduce immunogenicity and to make it function properly in a dog’s body. By converting the constant regions of the rat mAb to those of canine antibodies, a large part of the mAb is then of canine origin, while the variable regions remain intact, thus conserving the binding properties of the antibody. This chimeric mAb acquires effector functions which depend on the Fc region of the mAb, including antibody-dependent cell-mediated cytotoxicity and complement-dependent cytotoxicity. However, because those two functions are not required for a therapeutic anti-PD-L1 antibody, canine IgGD (equivalent to human IgG4) can be used as a heavy chain constant region to minimise possible side effects caused by the cytotoxicity of mAbs^16, 17^.

In this study, rat-dog chimeric anti-PD-L1 mAb was prepared and tested for its binding and blocking property *in vitro* using recombinant protein–based assays. Furthermore, the clinical efficacy of the chimeric mAb was evaluated in dogs with OMM or undifferentiated sarcoma, which might express PD-L1 as a possible mechanism of immune escape^13, 15^. Here we provide the first evidence of clinical benefit of anti-PD-L1 mAb in dogs, suggesting that it could be a novel option for canine cancer treatment.

## Results

### Selection of anti-PD-L1 antibody

**Figure 1:**
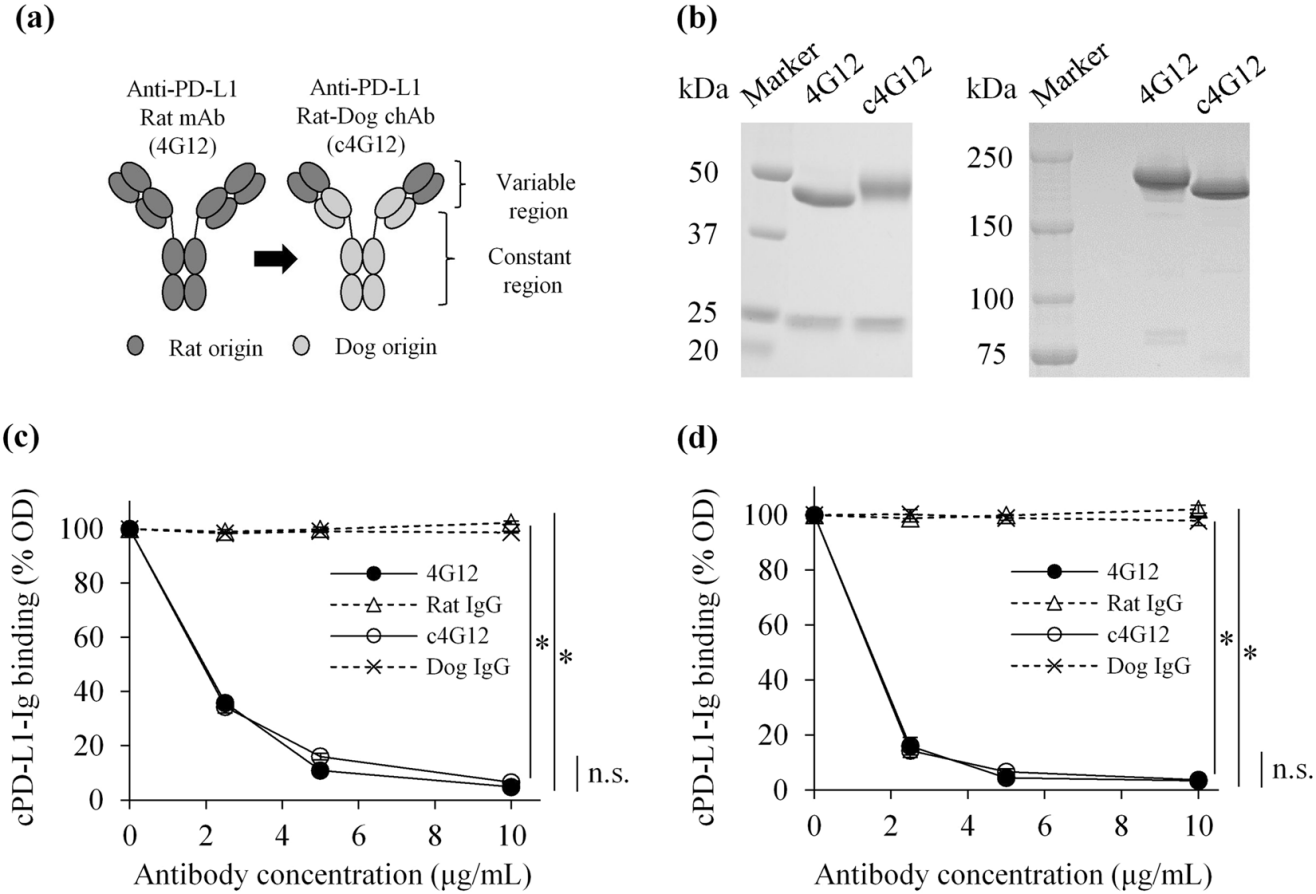
Preparation and evaluation of canine chimeric anti-PD-L1 mAb c4G12. (**a**) Schematic image of rat mAb and canine chimeric mAb. (**b**) Expression and purification of c4G12. CHO-DG44 cell lines which stably produce c4G12 were established and the culture supernatant was purified by protein A derivative. SDS-PAGE and Coomassie brilliant blue staining were performed and the images were analysed by densitometry to evaluate the protein purities. Left panel, reduced condition; Right panel, non-reduced condition. Rat mAb 4G12 was used as a control protein. Full-length gels are presented in Supplementary Figure S4. (**c**) Blocking of PD-1/PD-L1 binding by c4G12. cPD-1-Ig was coated on a microwell plate and binding of cPD-L1-Ig, which had been preincubated with various concentrations of anti-PD-L1 mAbs 4G12 or c4G12, was detected on the plate. Rat IgG and dog IgG were used as control antibodies. (**d**) Blocking of CD80/PD-L1 binding by c4G12. cCD80-Ig was coated on a plate and cPD-L1-Ig binding was evaluated as described above. Each point represents a mean value of relative OD (%) obtained from three independent experiments. Error bar; SE. Statistical analysis was performed by Tukey’s test. *p < 0.05; n.s., not significant.

To prepare therapeutic antibody for dog cancers, we compared three different rat anti-PD-L1 mAbs, that were recently established in our laboratory^14^. In the blocking assay of canine PD-1/PD-L1 binding, 6G7 (rat IgM) and 4G12 (rat IgG2a) showed efficient blocking ability, while 5A2 (rat IgG1) did not have an apparent blocking effect (Supplementary Fig. S1a). To reduce immunogenicity, rat antibody was canine-chimerised prior to clinical use (Fig. 1a). For this purpose, IgM antibody was converted to IgG in our chimeric mAb expression system, although the conversion between antibody classes may weaken binding avidity and blocking ability of the antibody. To assess this possibility, canine chimeric mAb, c4G12 or c6G7, was prepared using the variable regions of 4G12 or 6G7 and the constant regions of dog IgGD and the lambda chain. They were produced in a mammalian cell–based transient expression system and purified by affinity chromatography using proteinA derivative. The purities of these proteins were evaluated by SDS-PAGE and found to be <80%, thus they underwent further purification by gel filtration chromatography to obtain >90% purities (Supplementary Fig. S1b). In the blocking assay of PD-1/PD-L1 binding, the blocking ability of c6G7 was obviously weakened, whereas that of c4G12 remained sufficient throughout the conversion to the canine chimeric IgG form (Supplementary Fig. S1c). Therefore, c4G12 was selected as the therapeutic candidate and used in the following experiments.

### Canine chimeric 4G12 (c4G12) had similar binding and blocking properties to its original rat mAb

For the stable expression of c4G12, Chinese hamster ovary (CHO)-DG44 cells were transfected with the c4G12 expression vector and high-producer cell clones were established by the dihydrofolate reductase (DHFR)/methotrexate (MTX) method. The expression and purification of c4G12 was confirmed by SDS-PAGE analysis (Fig. 1b). The purity was routinely >90%, thus no further purification was needed. Because CD80/PD-L1 interaction also inhibits T-cell responses^18^, the blocking ability of c4G12 was tested in both a PD-1/PD-L1 binding assay and CD80/PD-L1 binding assay. c4G12 significantly blocked the binding of recombinant cPD-L1 to both cPD-1 (Fig. 1c) and cCD80 (Fig. 1d), and possessed comparable blocking ability to 4G12. In the surface plasmon resonance (SPR) analysis, c4G12 was bound to cPD-L1 with a similar binding property (K_D_ = 2.30 ± 0.07 nM, Table 1) to its original rat mAb 4G12. The K_D_ value of c4G12 was 10-fold smaller than that of cPD-1-Ig and cCD80-Ig, indicating that the binding avidity of c4G12 was practically higher than those of cPD-1-Ig and cCD80-Ig (Table 1). This result was consistent with the observation that c4G12 sufficiently blocked PD-1/PD-L1 and CD80/PD-L1 binding.

**Table 1:** Binding properties of mAbs and recombinant receptors to cPD-L1-His.

	k_a_ ( × 10^6^/Ms)	k_d_ ( × 10^−3^/s)	K_D_ (nM)
4G12	2.42 ± 0.10	4.54 ± 0.19	1.88 ± 0.06
c4G12	3.14 ± 0.19	7.19 ± 0.26	2.30 ± 0.07
cPD-1			25.4 ± 4.89
cCD80			24.3 ± 0.89

The equilibrium dissociation constant (K_D_) of 4G12 or c4G12 was determined by fitting with the 1:1 kinetic binding model, and that of cPD-1-Ig or cCD80-Ig was determined by fitting with the two state reaction model in SPR analysis. Data shown in the table are means ± SE of three independent experiments. k_a_, association rate constant; k_d_, dissociation rate constant.

### Cytokine production and lymphocyte proliferation were enhanced by c4G12

**Figure 2:**
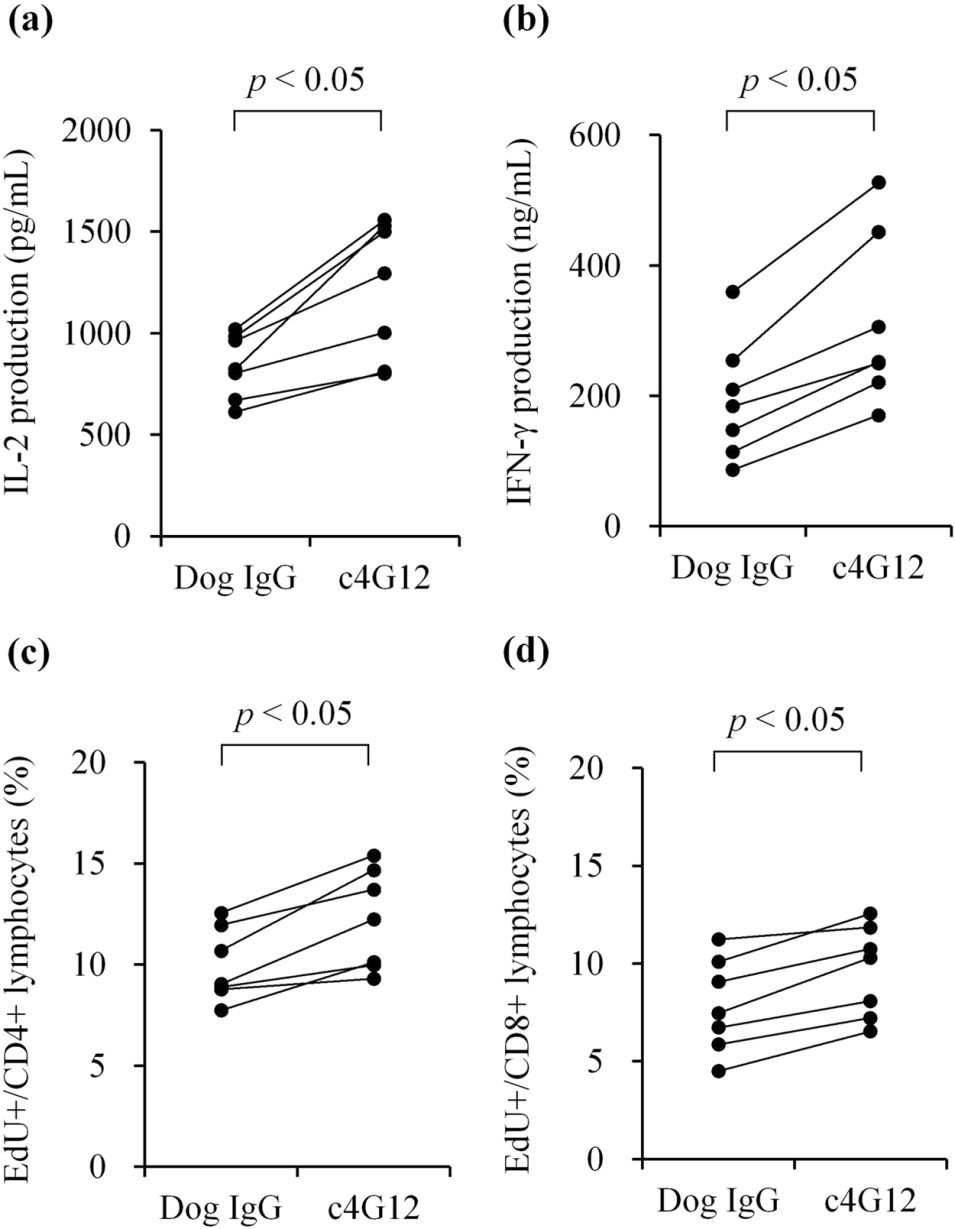
Enhancement of cytokine production and cell proliferation of dog peripheral blood mononuclear cells by c4G12 treatment. Dog peripheral blood mononuclear cells (*n* = 7) were obtained from healthy beagle donors and stimulated by 5 μg/mL staphylococcal enterotoxin B in the presence or absence of 20 μg/mL c4G12. Dog IgG was used as a control antibody. For evaluation of cytokine production, the culture supernatant was harvested on day 3, and concentration of (**a**) IL-2 or (**b**) IFN-γ was measured by ELISA. To evaluate cell proliferation, nucleotide analogue 5-ethynyl-2′-deoxyuridine (EdU) was added to the medium on day 2, and cells were harvested after incubation for another 2 h. The lymphocyte population was gated by forward scatter and side scatter, and the incorporation of EdU in (**c**) CD4+ or (**d**) CD8+ cells was measured by a flow cytometer. Statistical analysis was performed with a Wilcoxon signed rank-sum test.

To assess the immunomodulatory function of c4G12, dog peripheral blood mononuclear cells (PBMCs) were stimulated with the superantigen, staphylococcal enterotoxin B, in the presence or absence of c4G12. Cytokine production and cell proliferation were evaluated on days 3 and 2, respectively. Treatment with c4G12 significantly enhanced interleukin (IL)-2 and interferon (IFN)-γ production from dog PBMCs while cPD-L1-Ig treatment suppressed the production of IL-2 (Fig. 2a,b, Supplementary Fig. S2). The proliferation of CD4+ and CD8+ lymphocytes, indicated by the incorporation of nucleotide analogue 5-ethynyl-2′-deoxyuridine (EdU), was also enhanced by c4G12 treatment (Fig. 2c,d). Taken together, c4G12 appeared to restore the effector functions of dog lymphocytes which are suppressed by the PD-1/PD-L1 and/or CD80/PD-L1 axis.

### c4G12 treatment reduced tumour burden in dogs with oral malignant melanoma or undifferentiated sarcoma

**Figure 3:**
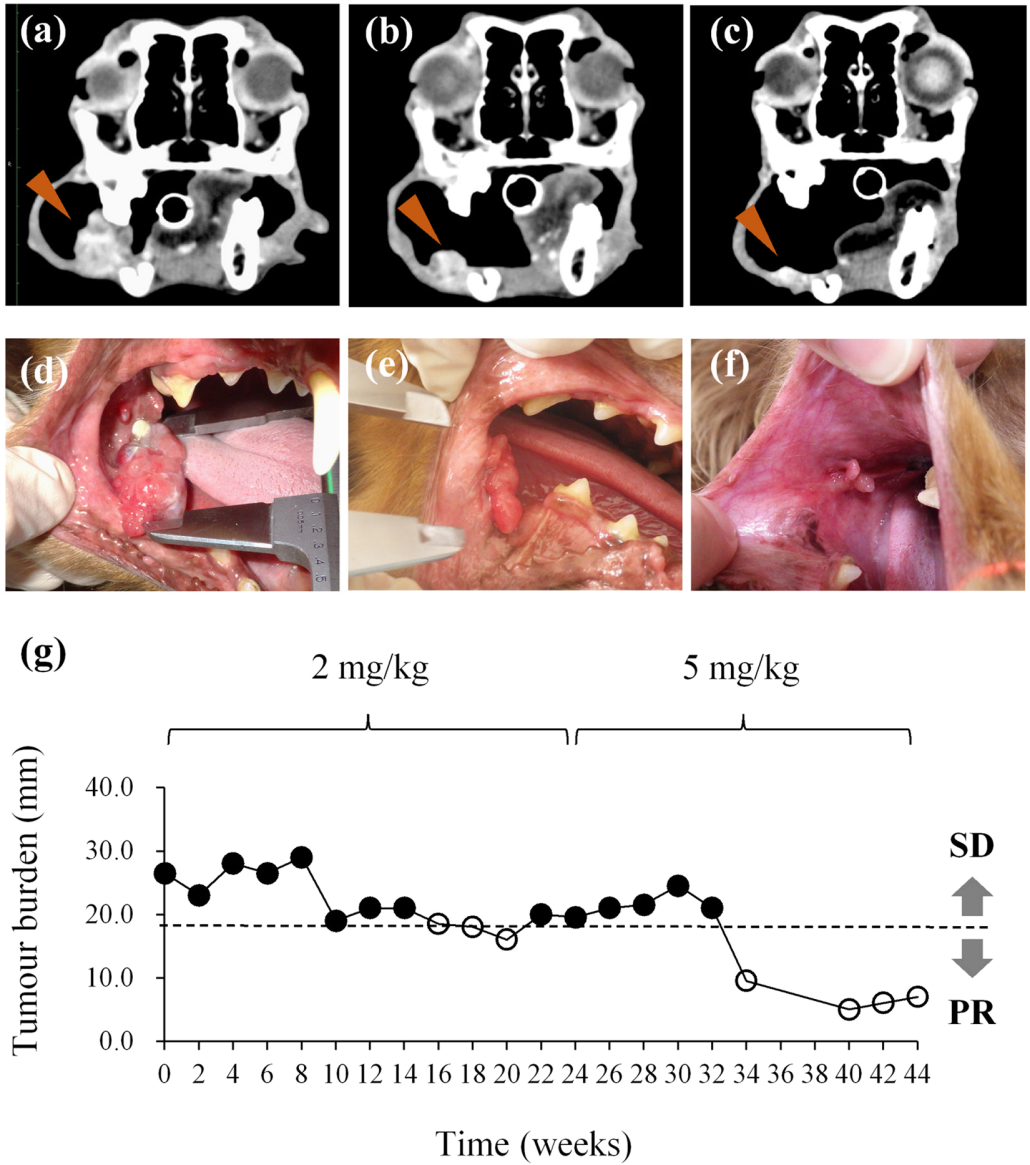
Antitumour effect of c4G12 in a dog with oral malignant melanoma. A dog with oral malignant melanoma was treated with 2 mg/kg (weeks 0–24) or 5 mg/kg (weeks 24–44) c4G12 every 2 weeks and the tumour burden was evaluated by gross examination and computed tomography (CT). (**a**,**d**) Tumour appearance at baseline (week 0). (**b**,**e**) Tumour appearance after 10 weeks of c4G12 treatment (**c**,**f**) Tumour appearance after 34 weeks of c4G12 treatment. Contrast-enhanced, and matched transverse CT images were shown. Arrowheads indicate the tumour lesions. (**g**) Changes in tumour burden. The longest diameter of target lesion was measured and recorded every 2 weeks. Reduction in tumour diameter by >30% (time points indicated by open circles) was considered PR (partial response; see Methods for definition of response).

**Figure 4:**
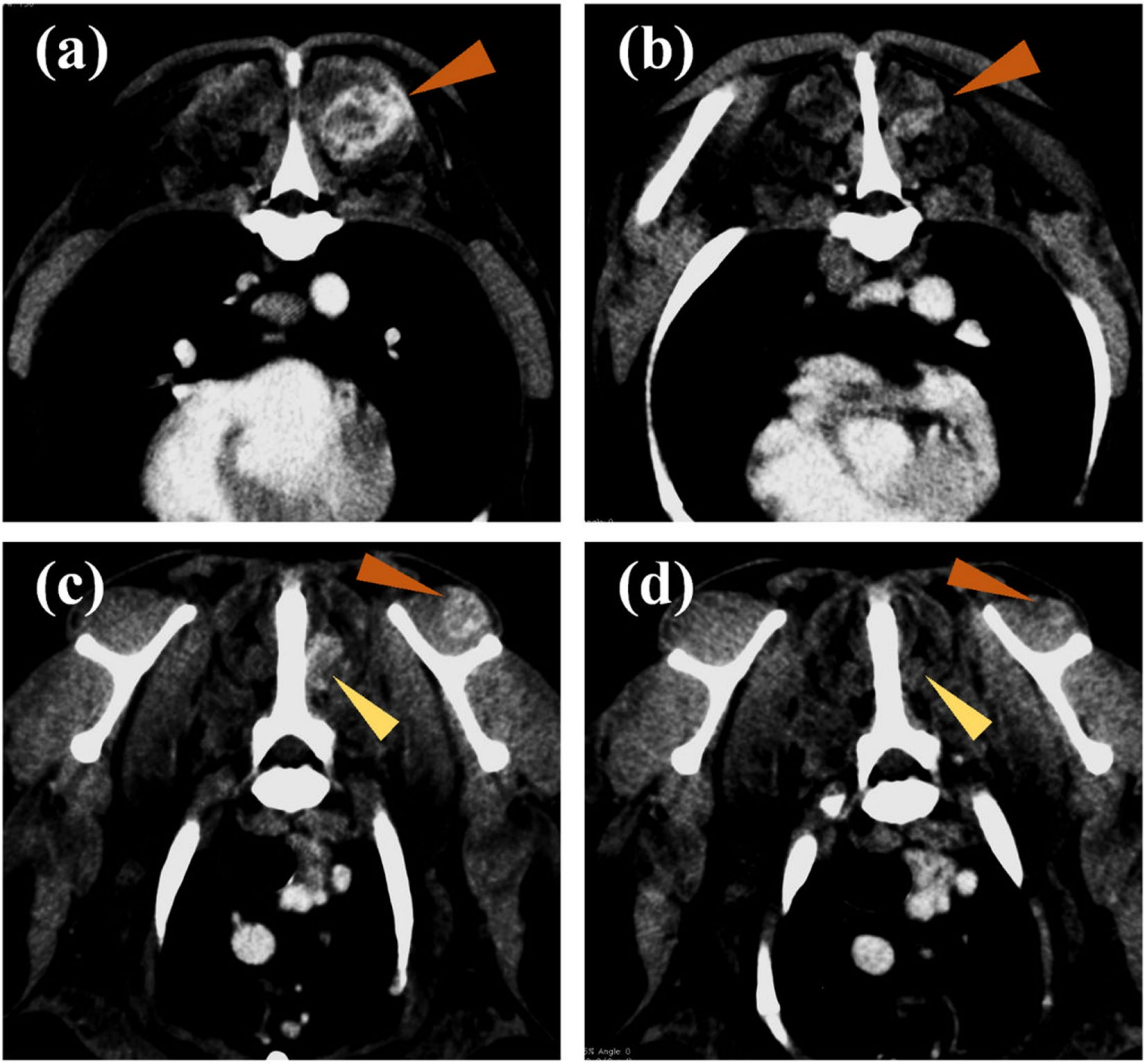
Antitumour effect of c4G12 in a dog with undifferentiated sarcoma. A dog with multiple metastatic lesions of undifferentiated sarcoma was treated with 5 mg/kg c4G12 every 2 weeks and the tumour burden was evaluated by computed tomography (CT). (**a**,**c**) Tumour appearance at baseline (week 0). (**b**,**d**) Tumour appearance after 3 weeks of c4G12 treatment. The tumours clearly responded to treatment on week 2, and the shrinkage was confirmed by CT on week 3. Contrast-enhanced, and matched transverse CT images were shown. Arrowheads indicate the tumour lesions.

Seven dogs histologically diagnosed with OMM and two dogs with undifferentiated sarcoma were enrolled in the pilot clinical trial, after the confirmation of PD-L1 expression in the primary cancers by immunohistochemistry (Supplementary Fig. S3). The dogs were treated with 2 or 5 mg/kg of c4G12 by intravenous administration every 2 weeks. Five miniature dachshunds, a pug, a golden retriever, a toy poodle and a West Highland white terrier were included, with a median age at the time of enrolment of 12 years (ranging from 11 to 16 years). Among dogs with OMM, one had stage II disease (primary tumour 2–4 cm in diameter, no lymph nodes involvement), two had stage III disease (lymph nodes involvement but without distant metastasis), and four had stage IV disease (with lung metastases) at the time of enrolment. Four dogs previously had surgery, and three had previous radiation therapy. During the antibody therapy, two dogs with lung metastases underwent concomitant palliative radiation therapy to control the primary oral cancers. Both dogs with undifferentiated sarcoma had multiple muscle metastases after surgical excision of primary tumours at the time of study enrolment (Table 2 and 3). In one dog (no. 1) with stage II OMM, obvious tumour regression was observed after 10 weeks of treatment with 2 mg/kg of c4G12 (Fig. 3a,b). However, the tumour began to grow slowly thereafter despite continuing treatment, thus the dose was increased to 5 mg/kg on week 24. Ten weeks after the dose change (week 34), the tumour again regressed dramatically (Fig. 3c) with only traces of the mass seen on gross examination and CT images (Fig. 3d–g, PR; approximately 81% reduction in the tumour burden at the maximum). In the other six dogs with OMM, the disease progressed with no evidence of antitumour response in detectable lesions (PD). The objective response rate (CR and PR) of OMM dogs was 14.3% (1/7). Among the two dogs with undifferentiated sarcoma, one (no. 9) clearly responded to c4G12 therapy after 3 weeks of treatment at 5 mg/kg (Fig. 4a,d), with an approximately 34% decrease in tumour burden (PR). The other dog with undifferentiated sarcoma had progression of the disease and dropped out of the study on week 3 (PD). The objective response rate of undifferentiated sarcoma dogs was 50.0% (1/2). No allergic reactions or autoimmune disease was seen throughout the trial, in which a total of 63 doses were given to nine dogs. No systemic toxicity was noted on routine physical examination, complete blood count or serum chemistry. Grade 1 diarrhoea was observed in a dog with OMM during treatment with 2 mg/kg (Table 3). It was transient and no medical intervention was needed. Although some other adverse events (mostly grade 1 or 2, changes in serum chemistry values *etc*.) were noted during the study, they were not considered treatment-related, and additional medication or cancellation of c4G12 treatment was not indicated.

**Table 2:** Characteristics of dogs treated with c4G12 in the pilot study.

Dog no.	Breed	Age (years)	Sex	Primary tumour	WHO stage for OMM	Prior therapy
1	Miniature dachshund	11	Male	Oral malignant melanoma	II	Surgery
2	Pug	11	Male, castrated	Oral malignant melanoma	IV	Surgery
3	Miniature dachshund	16	Female	Oral malignant melanoma	III	Radiation therapy
4	Miniature dachshund	14	Male, castrated	Oral malignant melanoma	IV	Radiation therapy
5	Golden Retriever	10	Male, castrated	Oral malignant melanoma	IV	Surgery
6	Miniature dachshund	14	Male	Oral malignant melanoma	IV	Radiation therapy
7	Miniature dachshund	14	Male, castrated	Oral malignant melanoma	III	None
8	Toy poodle	11	Male, castrated	Undifferentiated sarcoma	—	Surgery, Chemotherapy
9	West Highland white terrier	12	Male, castrated	Undifferentiated sarcoma	—	Surgery

Prior therapy included definitive/palliative surgery, definitive/palliative radiation therapy and chemotherapy with chlorambucil.

**Table 3:** Results of pilot study of dogs treated with c4G12.

Dog no.	Treatment duration (weeks)/c4G12 doses given	Dose of c4G12	Concomitant therapy	Best overall response	Survival after lung metastasis (days)	Treatment-related adverse event (VCOG-CTCAE)	Concurrent disease
1	44/22	Weeks 0–24: 2 mg/kg, then 5 mg/kg	None	PR	—	Diarrheoa, grade 1 (week 4)	None
2	32/16	Weeks 0–8: 2 mg/kg, then 5 mg/kg	Radiation therapy	PD	222	None	None
3	8/4	Weeks 0–2: 2 mg/kg, then 5 mg/kg	None	PD	—	None	None
4	6/3	2 mg/kg	None	PD	96	None	None
5	10/5	2 mg/kg	Radiation therapy	PD	91	None	None
6	12/6	5 mg/kg	None	PD	89	None	None
7	6/3	5 mg/kg	None	PD	—	None	None
8	3/2	5 mg/kg	None	PD	—	None	None
9	4/2	5 mg/kg	None	PR	—	None	Meningioma, Pulmonary fibrosis (suspected)

PR, partial response; PD, progressive disease. Adverse events which is possibly treatment-related are shown in the table.

### Prolongation of survival in dogs with oral malignant melanoma with pulmonary metastasis

**Figure 5:**
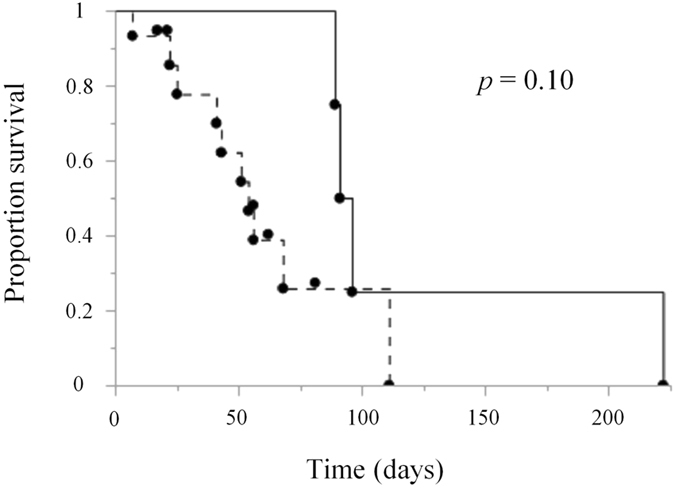
Survival in dogs with oral malignant melanoma after the confirmation of pulmonary metastasis. Survival (days) from the confirmation of pulmonary metastasis to death was recorded and plotted on the graph to generate Kaplan-Meier curves. The c4G12 treatment group (*n* = 4, black line) may have had prolonged survival compared to historical control group (*n* = 15, dashed line) treated by standard therapies at Veterinary Teaching Hospital during 2013 to 2016. Dots above the line indicate censored data (5 dogs in the control group were censored at 17, 21, 56, 62 and 81 days). Statistical analysis was performed with a log-rank test.

Among dogs with OMM treated with c4G12, four dogs with pulmonary metastasis had progression of the disease (PD). However, one dog had unexpectedly long survival (no. 2, 222 days) after the confirmation of pulmonary metastasis. Because reliable survival data for dogs with stage IV OMM (with confirmed distant metastasis) has not been reported in the literature to date, survival from the confirmation of pulmonary metastasis to death was retrospectively investigated in dogs with OMM treated at our veterinary hospital from 2013 to 2016, and the survival pattern of treatment group was compared to that of historical control group. The control group comprised nine miniature dachshunds, two Labrador retrievers, a Chihuahua, a golden retriever, a beagle, a Pomeranian, a miniature schnauzer and a mixed breed. The median age at the time of confirmation of pulmonary metastasis was 14 years (range, 10–16 years, *n* = 15). The treatment group comprised two miniature dachshunds, a pug and a golden retriever, with a median age at the time of confirmation of pulmonary metastasis of 12.5 years (range, 10–14 years, *n* = 4). The survival pattern of treatment group and that of control group was analysed by Kaplan-Meier method and the estimated median survival of treatment group and control group were 93.5 days (range, 89–220 days) and 54 days (range, 7–111 days), respectively (Fig. 5, *p* = 0.10).

## Discussion

Malignant melanoma is a cancer derived from melanocytes, which may occur in the oral cavity, lip, skin, digit or eye in dogs^19, 20^. In particular, OMM is highly invasive and metastatic^20^, making it difficult to cure the dog completely. When treated with surgery, median survival for dogs with OMM depend on the stage, as defined by the World Health Organization staging scheme. For stage I disease, survival is approximately 17 to 18 months, 5 to 6 months for stage II and 3 months for stage III^21^. Radiation therapy can be applied for local control of cancer in addition to or instead of surgery, but chemotherapy for systemic treatment of metastatic lesions in general yields insufficient tumour control. The lack of effective systemic therapy leads to a poor outcome in dogs with metastatic OMM, although the survival of dogs with stage IV OMM remains to be extensively studied. In this study, the survival of dogs with OMM with pulmonary metastasis was retrospectively investigated in our hospital and found to be less than 2 months (54 days). Development of alternative or additional systemic therapy is needed for the control of OMM, especially when it has metastasised.

Immunotherapy targeting immune checkpoint molecules such as PD-1 and PD-L1 has shown great promise for various cancer treatment in human and mouse studies^5, 22, 23^. However, in the field of veterinary science, little is known about the association between these molecules and animal diseases. We previously reported that PD-1 and PD-L1 were upregulated in chronic bovine infections such as bovine leukemia virus infection, Johne’s disease and bovine anaplasmosis, and the blockade of those molecules by specific mAbs associated with enhanced immune response^24–27^. In dogs, our group and others have reported PD-1 and PD-L1 expression in various cancer types, and the blockade by several mAbs restored the immune cell function *in vitro* ^13, 15, 28–31^. However, the therapeutic potential of those mAbs has not been reported *in vivo* because rat or mouse mAb may not be suitable for repeated administration to dogs for several reasons. First, because rodent protein itself is immunogenic for dogs, severe allergic reactions including anaphylaxis may occur. Second, an antibody response can be established against the administrated mAb itself, which may neutralise and reduce its therapeutic activity. To overcome these shortcomings, mAbs have been chimerised to prepare therapeutic antibodies for humans. For example, rituximab, anti-CD20 mAb for the treatment of non-Hodgkin’s lymphoma, is a mouse-human chimeric mAb and is widely used with tolerable side effect profiles^32^. Although humanised or fully humanised mAb has been developed and is considered a better option for therapeutic mAbs for humans, chimeric mAb is still a fascinating option for veterinary species because it does not need conformational analysis of animal antibodies or specialised mice genetically modified to produce animal antibodies. In fact, canine chimerisation of rat mAb was done quite simply in this study by combining the nucleotide sequence of the rat mAb variable regions with the dog mAb constant regions. Chimerisation did not affect the binding and blocking property of mAb when rat IgG was converted into dog IgG, and the chimeric mAb was well tolerated in dogs in the pilot clinical study, with no evidence of allergic reactions. Therefore, we concluded that chimerization of antibody is a simple and effective strategy to prepare therapeutic mAbs for dogs, although it should be validated in future studies in which a larger number of dogs or different chimeric mAbs are included in the evaluation.

To the best of our knowledge, this is the first report in which canine chimeric anti-PD-L1 mAb was prepared and tested for its clinical efficacy in dogs with malignant cancers. Treatment with c4G12 induced an obvious antitumour response in a dog with OMM and in another with undifferentiated sarcoma. The objective response rate in OMM was 14.3% (1/7) and that in undifferentiated sarcoma dogs was 50.0% (1/2). Because objective response rate of anti-PD-L1 mAb in melanoma patients was 17.3% (9/52) in a human clinical trial^22^, our result seems consistent with human studies. In addition, the c4G12 treatment may have prolonged survival in dogs with OMM with pulmonary metastasis, although statistical significance was not reached (*p* = 0.10), possibly because the sample size was too small (treatment group *n* = 4). Considering that dogs with OMM with pulmonary metastasis have quite a poor prognosis with standard therapies, c4G12 could be a novel treatment option for at least palliative purposes. Because the proof of concept has been established in this pilot clinical study, further clinical studies should be performed to fully elucidate the clinical benefit of c4G12.

In this study, dogs with PD-L1-positive cancers were selectively enrolled in the trial because, in a human clinical trial of anti-PD-1 mAb, 36% of patients with PD-L1-positive cancers responded to the treatment, whereas none with PD-L1-negative cancers had an objective response^5^. While both cell surface and intracellular expression of PD-L1 was observed in the tumour cells (Supplementary Fig. S3), any association between the staining pattern and clinical response was not noted. Although discussion continues as to whether measuring PD-L1 expression in cancers is a clinically useful predictive biomarker for response to PD-1/PD-L1 inhibitors^33^, other PD-L1-positive cancers in dogs including osteosarcoma, hemangiosarcoma, mast cell tumour, mammary adenocarcinoma, lymphoma and prostate adenocarcinoma^13, 15, 29, 31^, could also be targeted by c4G12. Clinical studies of c4G12 in these cancer types may be beneficial to find new treatment options for these highly malignant and refractory cancers.

Because no systemic toxicity or autoimmune disease was noted in the trial, c4G12 seems to be safe and well tolerated in dogs despite repeated administration. A mild diarrhoea was noted in one dog with OMM although a causal relationship with the treatment remains unclear (possibly treatment-related). In humans, side effects possibly caused by the PD-1/PD-L1 inhibiting–mAbs include infusion-related reactions, hypersensitivity, pneumonitis, colitis, diarrhoea and hypothyroidism^5, 12, 22^. Because evidence of autoimmune disease was reported in PD-1 knockout mice^34, 35^ and treatment-related deaths due to pneumonitis have been reported in a human clinical trial^5^, a careful attention should be paid to these possibilities in future studies in dogs using c4G12.

In conclusion, we have successfully developed a canine chimeric anti-PD-L1 mAb and tested its clinical efficacy in dogs with OMM or undifferentiated sarcoma. The pilot clinical study demonstrated the safety and antitumour activity of anti-PD-L1 mAb in dogs. Some dog cancers, including malignant melanoma, are considered preferable preclinical models of human cancer research with similar biological behaviours, resistance to chemo/radiotherapy, metastatic propensity and spontaneous occurrence in outbred and immunocompetent settings^36^. Therefore, further studies may be beneficial not only for developing dog cancer treatment but also to inform human preclinical studies using PD-1/PD-L1 inhibiting–mAbs.

## Methods

### Animals

Animal use throughout this study was approved by the Institutional Animal Care and Use Committee, Hokkaido University (Approval number: 15–0149) and the Graduate School of Veterinary Medicine, Hokkaido University (Approval number: 15028). All experiments were performed in accordance with relevant guidelines and regulations of the Graduate School of Veterinary Medicine, Hokkaido University, which has been fully accredited by the Association for Assessment and Accreditation of Laboratory Animal Care International (AAALAC). Peripheral blood samples of clinically healthy dogs were collected from 1- to 3-year-old beagles kept at the Experimental Animal Facility, Graduate School of Veterinary Medicine, Hokkaido University. Dogs with OMM or undifferentiated sarcoma seen at the Veterinary Teaching Hospital of Hokkaido University were enrolled in a pilot clinical study of the chimeric antibody. Written informed consent was obtained from the dogs’ owners and veterinarians.

### Expression and purification of canine chimeric mAb against dog PD-L1

To produce the canine chimeric anti-PD-L1 mAb 4G12 (c4G12), the expression vector, pDC6-c4G12, was prepared as described in the supplementary methods. For stable expression of chimeric mAb, pDC6-c4G12 was introduced into DHFR–deficient CHO-DG44 cells (Life Technologies, Carlsbad, CA, USA) using Lipofectamine LTX Reagent (Life Technologies) following the manufacturer’s instructions. Stable producer cells were selected in CD OptiCHO medium (Life Technologies) supplemented with 2 mM GlutaMAX I (Life Technologies). Cloning of expressing cells was performed following a standard protocol of limiting dilution. Gene amplification was performed by the DHFR/MTX method using CD OptiCHO medium supplemented with 2 mM GlutaMAX I and 60 nM MTX (Alexis Biochemicals, San Diego, CA, USA) in combination with further cell cloning. The culture supernatant of the expressing cells was harvested on day 14 of a shaking culture (37 °C, 5% or 8% CO_2_, 125 rpm) in CD OptiCHO medium or in Dynamis medium (Life Technologies) supplemented with 2 mM GlutaMAX I. For the culture in Dynamis medium, 4 g/L, 4 g/L and 6 g/L glucose was added on days 3, 5 and 7, respectively, and 3.3% v/v EfficientFeed B + (Life Technologies) reconstituted at 3 × concentration was added on days 3, 5, 7 and 10. Purification of chimeric mAb from the culture supernatant was performed by affinity chromatography using Ab-Capcher Extra (Protenova, Kagawa, Japan), and the buffer was exchanged with phosphate-buffered saline (PBS) by ultrafiltration using Amicon Ultra-15 Ultracel-50 (Merck Millipore, Billerica, MA, USA). The concentration of c4G12 was measured by Nanodrop 8000 Spectrophotometer (Thermo Fisher Scientific, Waltham, MA USA). To confirm the expression and purification of c4G12, SDS-PAGE was performed in reducing or non-reducing conditions using SuperSep Ace 5–20% gradient gel (Wako, Osaka, Japan) and 2× Laemmli Sample Buffer (Bio-Rad, Hercules, CA, USA). Precision Plus Protein All Blue Standards (Bio-Rad) was used as a molecular-weight size marker, and proteins were visualised with Quick-CBB (Wako). The purity of c4G12 was evaluated by densitometry using CS Analyzer version 3.0 software (Atto, Tokyo, Japan) and was routinely >90%. For SDS-PAGE analysis, rat anti-PD-L1 mAb 4G12 was used as the control protein.

### Blocking assay for PD-1/PD-L1 or CD80/PD-L1 binding

For the preparation of recombinant canine CD80 protein fused to rabbit IgG Fc (cCD80-Ig), the nucleotide sequence encoding the putative extracellular region of cCD80 was amplified by polymerase chain reaction (PCR) using cDNA obtained from beagle PBMCs stimulated with phorbol 12-myristate 13-acetate (20 ng/mL, Sigma-Aldrich, St. Louis, MO, USA) and ionomycin (1 μg/mL, Sigma-Aldrich) and the gene-specific primers (5′-CGC GGA TAT CAT GGA TTA CAC AGC GAA GTG-3′ and 5′-CGG GGT ACC CCA GAG CTG TTG CTG GTT AT-3′). The amplicon was then cloned into the multicloning site of the pCXN-2.1–rabbit IgG Fc vector (kindly provided by Dr T. Yokomizo, Juntendo University; modified)^37, 38^ using EcoRV and KpnI restriction enzyme sites. The resulting expression vector, pCXN2.1–rabbit IgG Fc-cCD80, was introduced into Expi293F cells (Life Technologies) and the recombinant protein was expressed according to the manufacturer’s instructions. cCD80-Ig was purified from culture supernatant harvested on days 2 and 7 using Ab-Capcher Extra (Protenova), and the buffer was exchanged with PBS using PD-MidiTrap G-25 (GE Healthcare UK, Buckinghamshire, UK). The concentration of cCD80-Ig was measured with a Pierce BCA Protein Assay Kit (Thermo Fisher Scientific).

To evaluate the ability of anti-PD-L1 antibody to block PD-1/PD-L1 or CD80/PD-L1 binding, a blocking assay was conducted on a microwell plate using recombinant canine PD-1, PD-L1 and CD80 proteins (cPD-1-Ig, cPD-L1-Ig and cCD80-Ig) as described in the supplementary methods. Briefly, a flat bottom microwell plate was coated with cPD-1-Ig or cCD80-Ig and blocked with PBS containing 1% bovine serum albumin and 0.05% Tween20. Biotinylated cPD-L1-Ig was preincubated with rat anti-PD-L1 mAb 4G12 or the canine chimeric mAb c4G12 at various concentrations (0, 2.5, 5, 10 μg/mL) for 30 min at 37 °C and added to the plate. cPD-L1-Ig binding was detected using avidin-horseradish peroxidase and tetramethylbenzidine substrate. Relative optic density (% OD) was calculated from the OD in comparison with that of control without antibody (0 μg/mL). Rat IgG (Sigma-Aldrich) or dog IgG (Jackson ImmunoResearch Laboratories, West Grove, PA, USA) was used as a negative control. Statistical analysis was performed by Tukey’s test among groups treated with 10 μg/mL of antibody. A *p* value of less than 0.05 was considered statistically significant.

### Measurement of binding avidity to canine PD-L1

To assess binding avidity of 4G12, c4G12, cPD-1-Ig and cCD80-Ig to canine PD-L1, SPR analysis was performed using polyhistidine-tagged canine PD-L1 protein (cPD-L1-His). For the preparation of cPD-L1-His, the nucleotide sequence of the putative extracellular region of canine PD-L1 was amplified by PCR as described previously^15^, except for the use of gene-specific primers containing the C-terminal 6 × polyhistidine-tag–encoding sequence (5′-CGC GGC TAG CAT GAG AAT GTT TAG TGT CTT-3′ and 5′-CGC GGA TAT CTT AAT GGT GAT GGT GAT GGT GAG TCC TCT CAC TTG CTG G-3′). The amplicon was then cloned into the multicloning site of the pCXN-2.1 vector (kindly provided by Dr T. Yokomizo, Juntendo University)^38^ using NheI and EcoRV restriction enzyme sites. The resulting expression vector, pCXN2.1–cPD-L1-His, was introduced into Expi293F cells (Life Technologies) and the recombinant protein was expressed according to the manufacturer’s instructions. cPD-L1-His was purified from culture supernatant harvested on days 2 and 7 using TALON Metal Affinity Resin (Clontech, Palo Alto, CA, USA), and the buffer was exchanged with PBS by ultrafiltration using Amicon Ultra-4 Ultracel-3 (Merck Millipore). The concentration of cPD-L1-His was measured with a Pierce BCA Protein Assay Kit (Thermo Fisher Scientific).

SPR analysis was performed using Biacore ×100 system with a CM5 sensor chip and His Capture Kit (GE Healthcare). Anti-histidine antibody was immobilised on the sensor chip by amine coupling following the manufacturer’s instructions. cPD-L1-His was captured on the sensor chip to obtain approximately 35 response units (RU) to analyse 4G12 or c4G12 binding, or approximately 400 RU to analyse cPD-1-Ig or cCD80-Ig binding. HBS-EP+ (GE Healthcare) was used for both the running and the dilution buffer. Control run responses containing buffer only were subtracted to obtain specific binding responses. The kinetic constants of 4G12 or c4G12 were determined by fitting with the 1:1 kinetic binding model, and that of cPD-1-Ig or cCD80-Ig was determined by fitting with the two state reaction model because a conformational change of PD-L1 upon binding with PD-1 and CD80 has been suggested^39^.

### *In vitro* functional assay of anti-PD-L1 chimeric mAb

PBMCs were prepared from healthy beagles and cultured as described previously^15^, in the presence of 5 μg/mL staphylococcal enterotoxin B (Sigma-Aldrich) and 20 μg/mL c4G12. Dog IgG (Jackson ImmunoResearch Laboratories) was used as a control antibody. For cytokine assays, the culture supernatant was harvested on day 3 and the concentrations of IL-2 and IFN-γ were measured by Duoset ELISA canine IL-2 and IFN-γ (R&D systems, Minneapolis, MN, USA), respectively, according to the manufacturer’s instructions. For the proliferation assay, incorporation of nucleotide analogue was evaluated using a Click-iT Plus EdU flow cytometry assay kit (Life Technologies). The thymidine analogue, EdU, was added to the culture medium at a final concentration of 10 μM on day 2, and cells were harvested after additional incubation for 2 h. Cells were stained with optimal concentrations of anti-canine CD4 antibody (R&D systems) conjugated with PerCP-Cy5.5 using a Lightning-Link Antibody Labeling Kit (Innova Biosciences, Cambridge, UK) and anti-canine CD8 antibody (LifeSpan BioSciences, Seattle, WA, USA) labelled with R-PE using a Zenon Mouse IgG2a Labeling Kit (Thermo Fisher Scientific). After washing, incorporated EdU was labelled with Alexa Fluor 647 following the manufacturer’s instructions. Fluorescence of the cells was analysed with a FACS Verse flow cytometer (BD Biosciences, San Jose, CA, USA). For the analysis, the lymphocyte population was gated using forward scatter and side scatter, and EdU incorporation in CD4+ cells or CD8+ cells was evaluated within the lymphocyte population. A Wilcoxon signed rank-sum test was performed to compare data obtained from the same individuals. The result was considered statistically significant if the *p* value was less than 0.05.

### Preparation of c4G12 for the clinical trial

Antibodies used in clinical studies must be highly purified to maximise the therapeutic potency and to avoid possible side effects. To reduce impurities and aggregates, culture supernatant containing c4G12 was obtained as described above and purified in a series of steps. First, c4G12 was purified from the supernatant by affinity chromatography using a HiScale 26/20 column packed with MabSelect SuRe LX (GE Healthcare). Additional purification by hydroxyapatite chromatography was performed using a BioScale CHT20-I prepacked column (Bio-Rad), and fractions containing aggregates were further purified by anion exchange chromatography using a HiScreen Q-Sepharose HP prepacked column (GE Healthcare). Throughout the purification steps, an ÄKTA avant 150 chromatography system (GE Healthcare) was used. The buffer was exchanged with PBS using a Vivaspin20 concentrator with 50 kDa molecular weight cut of membrane (Sartorius, Gӧttingen, Germany) and stored at 4 °C until use.

### Clinical trial on dogs with oral malignant melanoma or undifferentiated sarcoma

Before starting the clinical trial, the safety of c4G12 was confirmed in an experimental dog (13-year-old, female, beagle) by inoculating c4G12 intravenously at 2 mg/kg, every 2 weeks, 3 times in total. No allergic reactions or acute toxicity was found during 2 months of observation period. Therefore, to evaluate the clinical efficacy of c4G12, a pilot clinical study was conducted at Veterinary Teaching Hospital, Hokkaido University. The study period was from March 2016 to January 2017. Dogs with OMM or undifferentiated sarcoma were enrolled in the study if the expression of PD-L1 was confirmed in the cancer cells. The expression of PD-L1 in the primary tumour sections, obtained by surgical excision at prior surgery or biopsy, was assessed by immunohistochemistry as described elsewhere using anti-bovine PD-L1 mAb^13, 15^. Dogs treated with previous therapies such as surgery, radiation therapy or chemotherapy were included, while dogs with severe systemic illness or autoimmune disease were excluded from the study. c4G12 was diluted in 50 or 100 mL of saline depending on the body weight of dog (100 mL of saline was used if >20 kg) and administrated at 2 or 5 mg/kg, every 2 weeks, intravenously using a syringe pump over 1 h. During the treatment period, the dogs were monitored with physical examination, complete blood count and serum chemistry (including Glucose, ALT, ASP, ALP, LDH, CRP and T4) at least every 2 weeks. The tumour size was measured by a caliper and recorded every 2 weeks if measurable lesions were present on the body surface. At baseline (within 2 weeks prior to the first c4G12 administration) and every 6 weeks during treatment, computed tomography (CT) was performed to evaluate the tumour burden and metastases. For dogs with OMM, TNM-based staging was performed using the World Health Organization staging scheme^40^ at the time of study enrolment by baseline CT and fine-needle aspiration/cytology of lymph nodes when required. The tumour burden was calculated as the sum of the longest diameters of all target measurable lesions. Tumours ≥10 mm in the longest diameter were considered as measurable lesions. A maximum of five target lesions were chosen from measurable lesions at baseline, with a maximum of two lesions per organ. Tumour response to c4G12 treatment was defined as follows: complete response (CR), disappearance of all detectable tumour; partial response (PR), at least 30% reduction in the sum of the long diameters of target lesions; stable disease (SD), less than 20% increase or 30% reduction in the sum of diameters for at least 6 weeks and progressive disease (PD), at least 20% increase in the sum of diameters. The longest diameters of new measurable lesions (up to five lesions in total and up to two lesions per organ) were included in the sum. Unequivocal progression of nontarget lesions was considered PD (according to modified criteria from response evaluation criteria in solid tumours in dogs v 1.0^41^ and unidimensional immune-related response criteria^42, 43^). If concomitant radiation therapy was given for local control, the lesions targeted by radiation were excluded from the response evaluation to avoid overestimation. No potentially immunosuppressive medications (i.e., cytotoxic chemotherapy or immunosuppressive doses of steroids) were given concomitantly with the chimeric mAb treatment. Nonimmunosuppressive drugs, including NSAIDs, antibiotics, anodynes and antitussives were allowed for use during the study where clinically necessary. Adverse events were graded and recorded according to the veterinary cooperative oncology group – common terminology criteria for adverse events (VCOG-CTCAE) v 1.1^44^.

### Analysis of survival duration after confirmation of pulmonary metastasis

Survival from the confirmation of pulmonary metastasis to death was retrospectively investigated in dogs with OMM treated at the Veterinary Teaching Hospital, Hokkaido University from 2013 to 2016. The dogs treated with standard therapy, such as definitive/palliative surgery, definitive/palliative radiation therapy or chemotherapy using cyclophosphamide/carboplatin at any time from diagnosis to death were included (*n* = 15, control group). Among the dogs enrolled in the pilot clinical study, all four dogs with stage IV disease had pulmonary metastases (*n* = 4, treatment group). Kaplan-Meier method was used to estimate the median survival of control group and treatment group. Kaplan-Meier curves were generated using statistical analysis software JMP12 (SAS Institute, Cary, NC, USA), and a log-rank test was used to compere the survival patterns. A *p* value of less than 0.05 was considered statistically significant.

## Electronic supplementary material

Supplementary Information
