# Supplementary material for: A canine chimeric monoclonal antibody targeting PD-L1 and its clinical efficacy in canine oral malignant melanoma or undifferentiated sarcoma
Source: Sci Rep. 2017 Aug 21;7:8951. doi: 10.1038/s41598-017-09444-2 (PMC5567082; doi:10.1038/s41598-017-09444-2)
Supplement: Supplementary file 1 — Supplementary Information [file 41598_2017_9444_MOESM1_ESM.pdf]

A canine chimeric monoclonal antibody targeting PD-L1 and its clinical efficacy in canine oral malignant melanoma or undifferentiated sarcoma

Naoya Maekawa, Satoru Konnai, Satoshi Takagi, Yumiko Kagawa, Tomohiro Okagawa, Asami Nishimori, Ryoyo Ikebuchi, Yusuke Izumi, Tatsuya Deguchi, Chie Nakajima, Yukinari Kato, Keiichi Yamamoto, Hidetoshi Uemura, Yasuhiko Suzuki, Shiro Murata, Kazuhiko Ohashi

Supplementary methods

Preparation of canine chimeric monoclonal antibody (mAb) by a mammalian transient expression system

The Rapid Amplification of cDNA Ends (RACE) method was performed to determine the nucleotide sequence of rat anti-PD-L1 mAb 4G12 or the 6G7[14] variable region using a 5´-RACE system (Life Technologies, Carlsbad, CA, USA) following the manufacturer's instruction. Total RNA was extracted from the hybridoma 4G12 or 6G7 and the following rat immunoglobulin gene-specific primers were used: 5´-ACA AGG ATT GCA TTC CCT TGG-3´ and 5´-CTC AAT TTT CTT GTC CAC CTT GGT GC-3´ for 4G12 heavy chain; 5´-CAC ATT GGG GTT CAT CTC AAC-3´ and 5´-TGG AAT CGG CAC ATG CAG ATC T-3´ for 6G7 heavy chain; 5´-CTC ATT CCT GTT GAA GCT CTT GAC GAC-3´ and 5´-CTC ATT CCT GTT GAA GCT CTT GAC GAC GGG-3´ for 4G12 and 6G7 light chains. The amplicons were analyzed with a CEQ8000 DNA analysis system (Beckman Coulter) as described previously[15]. The nucleotide sequences of the light and heavy chain variable region were combined with those of dog lambda chain constant region (Genbank accession number E02824) and dog IgGD constant region (Genbank accession number AF354267), respectively, using Mega 5 software[45]. The resulting sequences were modified according to the optimal codon usage for expression in Chinese hamster ovarian cells (except for the signal peptide sequences, which were optimised for expression in *Pichia pastoris*), synthesized (Medical & Biological Laboratories, Aichi, Japan), and cloned into pDC6 expression vector (kindly provided by Dr. Y. Suzuki, Hokkaido University). The expression vector was named pDC6-c4G12 (canine chimeric 4G12) or pDC6-c6G7.

Expression vector for c4G12 or c6G7 were transfected into Expi293F cells (Life Technologies) and chimeric mAbs were produced according to the manufacturer's instructions. Culture supernatant was harvested on days 2 and 7. Chimeric mAbs were purified from culture supernatant by affinity chromatography using Ab-Capcher Extra (Protenova, Kagawa, Japan), and the buffers were exchanged with phosphate-buffered saline (PBS) using PD-MidiTrap G-25 (GE Healthcare UK, Buckinghamshire, UK). Concentrations of chimeric mAbs were measured by a Nanodrop 8000 Spectrophotometer (Thermo Fisher Scientific, Waltham, MA, USA). To confirm the expression and purification of chimeric mAbs, SDS-PAGE was performed in a non-reducing condition using 10% acrylamide gel and 2× Laemmli Sample Buffer (Bio-Rad, Hercules, CA, USA). Precision Plus Protein All Blue Standards (Bio-Rad) was used as a molecular-weight size marker and proteins were visualized with Quick-CBB (Wako). Purities of chimeric mAbs were evaluated by densitometry using CS Analyzer version 3.0 software (Atto, Tokyo, Japan). For further purification, gel filtration chromatography was performed using a Hiload 16/60 Superdex 200 pg (GE Healthcare) prepacked column and an ÄKTAexplorer FPLC system (Amersham Biosciences, Piscataway, NJ, USA). Rat mAb 4G12 and dog IgG (Jackson ImmunoResearch Laboratories, West Grove, PA, USA) were used as control proteins.

Blocking assay of PD-1/PD-L1 binding

To evaluate the ability of anti-PD-L1 mAb to block PD-1/PD-L1 binding, a blocking assay was conducted on a microwell plate using recombinant canine PD-1 and PD-L1 proteins (cPD-1-Ig and cPD-L1-Ig). The expression vector for cPD-1-Ig or cPD-L1-Ig (pCXN2.1–rabbit IgG Fc-cPD-1 or pCXN-2.1–rabbit IgG Fc-cPD-L1)[15] was introduced into Expi293F cells (Life technologies) and the recombinant proteins were expressed according to the manufacturer's instructions. cPD-1-Ig and cPD-L1-Ig were purified from culture supernatant harvested on days 2 and day 7 using Ab-Capcher Extra (Protenova, Kagawa, Japan), and the buffers were exchanged with PBS using PD-MidiTrap G-25 (GE Healthcare UK). Concentration of cPD-1-Ig or cPD-L1-Ig was measured by Pierce BCA Protein Assay Kit (Thermo Fisher Scientific). cPD-L1-Ig was biotinylated using Lightning-Link Biotin Conjugation Kit (Innova Biosciences, Cambridge, UK). A Maxisorp flat bottom microwell plate (Thermo Fisher Scientific) was coated with cPD-1-Ig and blocked with PBS containing 1% bovine serum albumin (Sigma-Aldrich, St. Louis, MO, USA) and 0.05% Tween20 (Kanto Chemical, Tokyo, Japan). Biotinylated cPD-L1-Ig was preincubated with anti-PD-L1 antibody 4G12, 5A2, 6G7[14], c4G12 or c6G7 at various concentrations (0, 2.5, 5, 10 μg/mL) for 30 min at 37 °C and added to the plate. cPD-L1-Ig binding was detected using Neutravidin-horseradish peroxidase (Thermo Fisher Scientific) and TMB one component substrate (Bethyl Laboratories, Montgomery, TX, USA). The reaction was stopped by adding 0.18 M H_2SO_4, and the optical density (OD) at 450 nm was measured by a microplate reader MTP-900 (Corona Electric, Ibaraki, Japan). Relative OD (% OD) was calculated from the OD in comparison with that of control without antibody (0 μg/mL). Rat IgG (Sigma-Aldrich), rat IgM (BD Biosciences, San Jose, CA, USA), or dog IgG (Jackson ImmunoResearch) was used as negative control. Statistical analysis was performed by Tukey's test among groups treated with 10 μg/mL of antibody. A p value of less than 0.05 was considered statistically significant.

In vitro functional assay of cPD-L1-Ig

Peripheral blood mononuclear cells were prepared from healthy beagles and cultured as described previously[15], in the presence of 5 μg/mL staphylococcal enterotoxin B (Sigma-Aldrich) and 100 nM of cPD-L1-Ig. Rabbit IgG (Southern Biotech, Birmingham, AL, USA) was used as negative control protein. The culture supernatant was harvested on day 3, and the concentrations of interleukin-2 were measured by Duoset ELISA canine IL-2 (R&D systems, Minneapolis, MN, USA) according to the manufacturer's instructions. A Wilcoxon signed rank-sum test was performed to compare the data obtained from the same individuals. The result was considered statistically significant if the p value was less than 0.05.

Supplementary figures

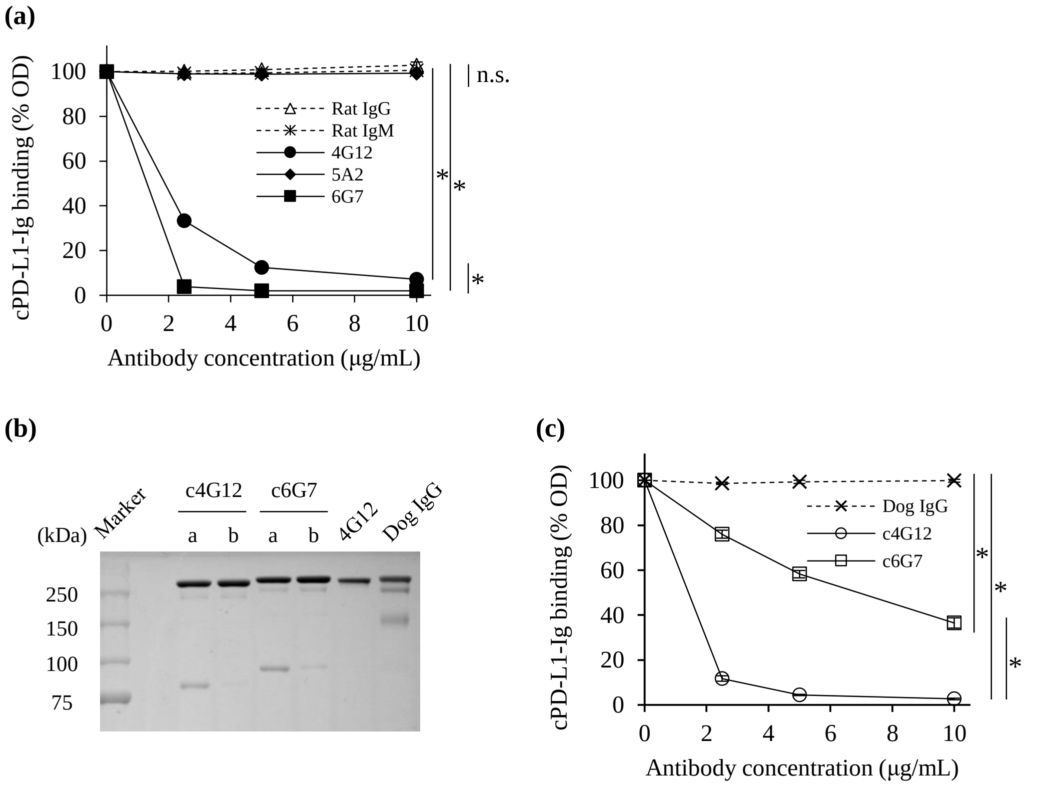

Supplementary Figure S1. Selection of anti-PD-L1 mAb for the preparation of canine chimeric mAb as a therapeutic candidate.
(a) Blocking effect of anti-PD-L1 mAbs. cPD-1-Ig was coated on a microwell plate and the binding of cPD-L1-Ig, which had been preincubated with various concentrations of anti-PD-L1 mAbs 4G12, 5A2, and 6G7, was detected on the plate. Rat IgG and rat IgM were used as control antibodies. (b) Expression and purification of canine chimeric 4G12 (c4G12) and c6G7. Chimeric mAbs were expressed by Expi293F cells and purified from culture supernatant by a) protein A derivative or b) protein A derivative plus gel filtration chromatography. SDS-PAGE and CBB staining were performed and the images were analysed by densitometry to evaluate the protein purity. Rat mAb 4G12 and dog IgG were used as control proteins. The full-length gel is presented in Supplementary Figure S4. (c) Blocking effects of chimeric mAbs c4G12 and c6G7. cPD-L1 binding to cPD-1-Ig was assessed similarly after preincubation with chimeric mAbs. Dog IgG was used as a control antibody. Each point represents a mean value of relative OD (%) obtained from three independent experiments. Error bar; SE. Statistical analysis was performed by Tukey's test. *$p < 0.05$; n.s., not significant.

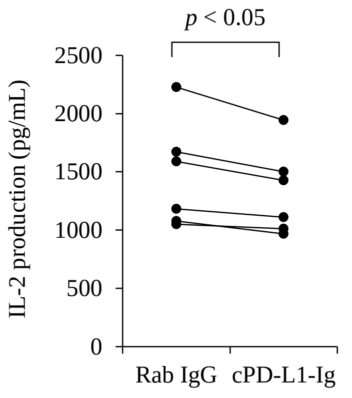

Supplementary Figure S2. Suppression of interleukin-2 production from dog peripheral blood mononuclear cells by cPD-L1-Ig treatment.

Peripheral blood mononuclear cells were obtained from healthy beagle donors (*n* =7) and stimulated by 5 μg/mL staphylococcal enterotoxin B in the presence or absence of 100 nM cPD-L1-Ig. The culture supernatant was harvested on day 3 and concentration of interleukin (IL)-2 was measured by ELISA. Rabbit IgG was used as negative control protein. Statistical analysis was performed with a Wilcoxon signed rank-sum test.

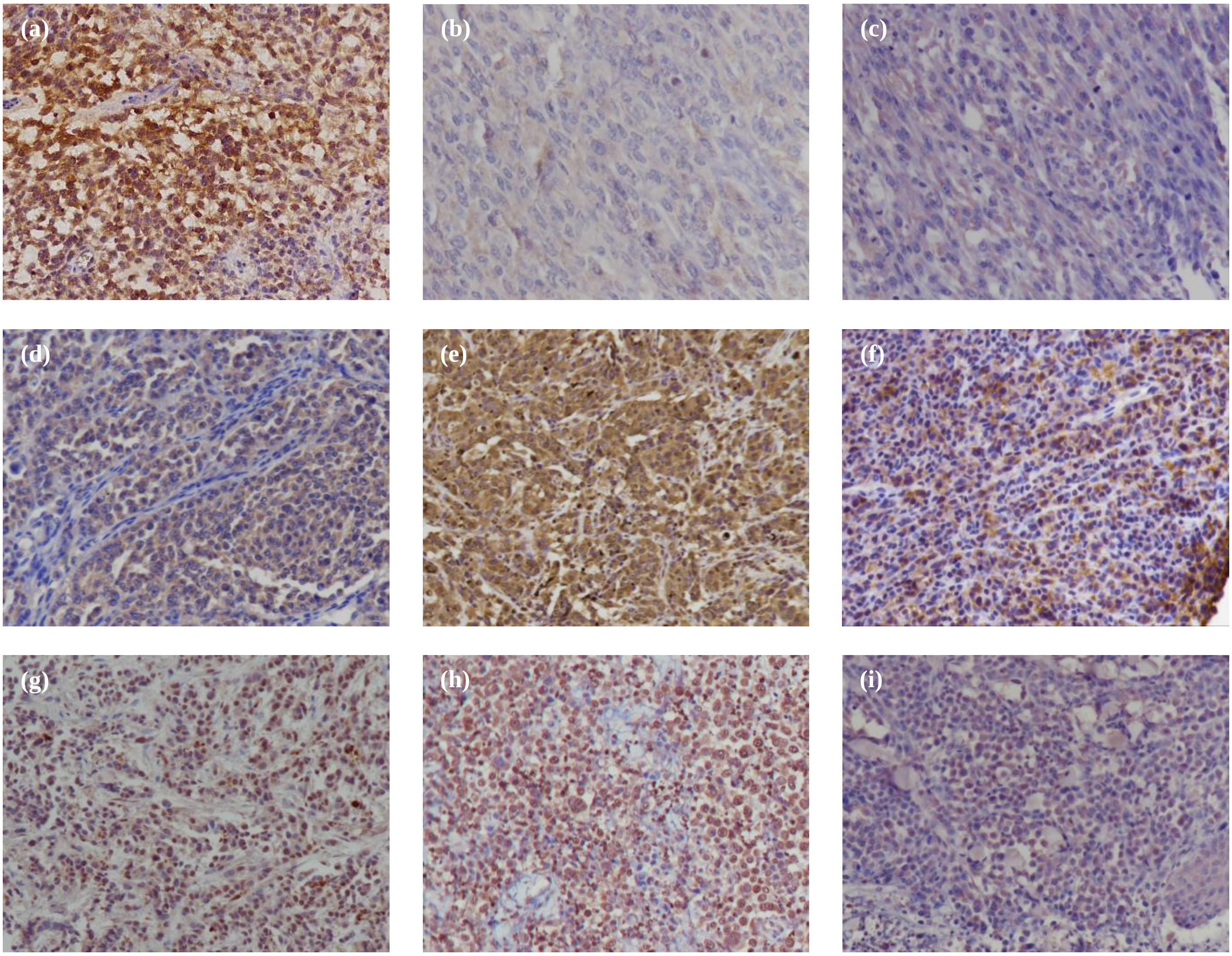

Supplementary Figure S3. Expression of PD-L1 in tumour tissues from dogs enrolled in the pilot clinical trial of c4G12.

Tumour sections obtained by surgical excision at prior surgery or biopsy were tested for PD-L1 expression by immunohistochemistry. (a-g) PD-L1 expression in oral malignant melanoma tissue obtained from dog no. 1-7, respectively. (h,i) PD-L1 expression in undifferentiated sarcoma tissue obtained from dog no. 8 and 9, respectively.

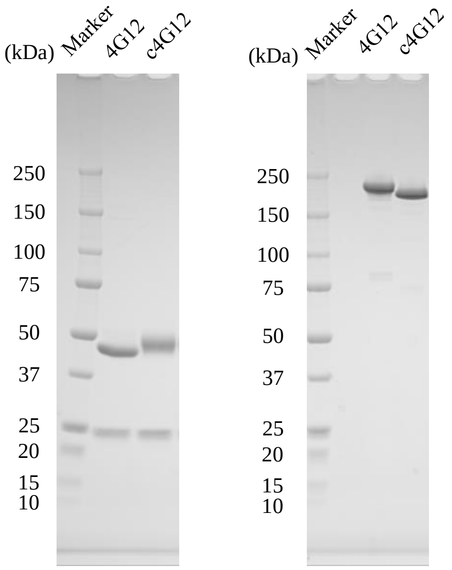

Fig. 1b

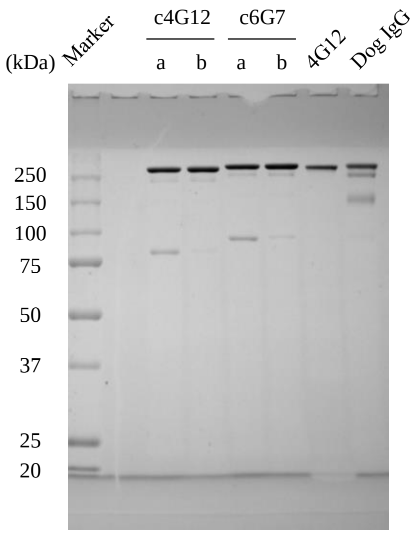

Supplementary Fig. S1b

Supplementary Figure S4. Full-length gels for Fig. 1b and Supplementary Fig. S1b.
